# Peer support for people with severe mental illness versus usual care in high-, middle- and low-income countries: study protocol for a pragmatic, multicentre, randomised controlled trial (UPSIDES-RCT)

**DOI:** 10.1186/s13063-020-4177-7

**Published:** 2020-05-01

**Authors:** Galia S. Moran, Jasmine Kalha, Annabel S. Mueller-Stierlin, Reinhold Kilian, Silvia Krumm, Mike Slade, Ashleigh Charles, Candelaria Mahlke, Rebecca Nixdorf, David Basangwa, Juliet Nakku, Richard Mpango, Grace Ryan, Donat Shamba, Mary Ramesh, Fileuka Ngakongwa, Alina Grayzman, Soumitra Pathare, Benjamin Mayer, Bernd Puschner

**Affiliations:** 1 The Charlotte B. and Jack J. Spitzer Department of Social Work, Ben Gurion University of the Negev, Be’er Sheva, Israel; 2 Centre for Mental Health Law and Policy, Indian Law Society, Pune, India; 3 Department of Psychiatry and Psychotherapy II, Ulm University, Ulm, Germany; 4 School of Health Sciences, Institute of Mental Health, University of Nottingham, Nottingham, UK; 5 Department of Psychiatry, University Medical Centre Hamburg-Eppendorf, Hamburg, Germany; 6 Butabika National Referral Hospital, Kampala, Uganda; 7 Centre for Global Mental Health, London School of Hygiene and Tropical Medicine, London, UK; 8 Ifakara Health Institute, Dar es Salaam, Tanzania; 9 Institute for Medical Biometry and Epidemiology, Ulm University, Ulm, Germany

**Keywords:** Peer support, Severe mental illness, Pragmatic randomised controlled trial, Process evaluation, Cost-effectiveness analysis, Implementation science, Global mental health

## Abstract

**Background:**

Peer support is an established intervention involving a person recovering from mental illness supporting others with mental illness. Peer support is an under-used resource in global mental health. Building upon comprehensive formative research, this study will rigorously evaluate the impact of peer support at multiple levels, including service user outcomes (psychosocial and clinical), peer support worker outcomes (work role and empowerment), service outcomes (cost-effectiveness and return on investment), and implementation outcomes (adoption, sustainability and organisational change).

**Methods:**

UPSIDES-RCT is a pragmatic, parallel-group, multicentre, randomised controlled trial assessing the effectiveness of using peer support in developing empowering mental health services (UPSIDES) at four measurement points over 1 year (baseline, 4-, 8- and 12-month follow-up), with embedded process evaluation and cost-effectiveness analysis. Research will take place in a range of high-, middle- and low-income countries (Germany, UK, Israel, India, Uganda and Tanzania). The primary outcome is social inclusion of service users with severe mental illness (*N* = 558; *N* = 93 per site) at 8-month follow-up, measured with the Social Inclusion Scale. Secondary outcomes include empowerment (using the Empowerment Scale), hope (using the HOPE scale), recovery (using Stages of Recovery) and health and social functioning (using the Health of the Nations Outcome Scales). Mixed-methods process evaluation will investigate mediators and moderators of effect and the implementation experiences of four UPSIDES stakeholder groups (service users, peer support workers, mental health workers and policy makers). A cost-effectiveness analysis examining cost-utility and health budget impact will estimate the value for money of UPSIDES peer support.

**Discussion:**

The UPSIDES-RCT will explore the essential components necessary to create a peer support model in mental health care, while providing the evidence required to sustain and eventually scale-up the intervention in different cultural, organisational and resource settings. By actively involving and empowering service users, UPSIDES will move mental health systems toward a recovery orientation, emphasising user-centredness, community participation and the realisation of mental health as a human right.

**Trial registration:**

ISRCTN, ISRCTN26008944. Registered on 30 October 2019.

## Background

Peer support is part of a broader recovery agenda in mental health that places emphasis on user-centred outcomes such as social inclusion and empowerment [1]. Peer support is an established intervention whereby a person in recovery from mental illness offers support to others living with mental illness [2]. Peer support workers (PSWs) support their own recovery and the recovery of others by drawing on their lived experiences, employing positive self-disclosure, expanding social networks, and promoting hope, empowerment and self-efficacy. Around the world, diverse PSW roles have been developed and formalised: peer companions, peer advocates, consumer case managers, peer specialists, peer counsellors and more [3]. PSWs offer a wide range of services, which may include social support, management of symptoms, counselling, outreach, coaching and advocacy [4]. Peer support can also be provided in different settings—as an alternative to, an independent service within, or an integral part of professional care [5].

Findings from meta-analyses of controlled studies indicate that PSWs are able to achieve outcomes comparable to professionally trained staff, and might therefore represent cost-effective additions to task-sharing models in low- and middle-income countries as well as in high-income countries [6, 7]. Qualitative and quantitative studies have also shown that peer support has a positive impact on recovery-related outcomes that may not be achieved using clinical interventions alone [8, 9]. However, there are significant gaps in the evidence base for peer support in low- and middle-income countries [10] and in non-Anglophone countries [11]. Most reviews of the literature on peer support have identified only studies from high-income countries, and these are primarily from English-speaking parts of the world [12].

### The UPSIDES consortium

Using Peer Support in Developing Empowering mental health Services (UPSIDES) investigates the effectiveness and implementation of peer support in a range of high-, middle- and low-income countries to generate evidence on a scalable model of recovery-oriented mental health care that may be transferable to similar settings. UPSIDES is a research consortium involving eight collaborating institutions across six countries: Ulm University, Germany; University of Nottingham, UK; University Hospital Hamburg-Eppendorf, Germany; Butabika National Referral Hospital, Kampala, Uganda; London School of Hygiene and Tropical Medicine, UK; Ifakara Health Institute, Dar es Salaam, Tanzania; Ben-Gurion University of the Negev, Be’er Sheva, Israel; and Centre for Mental Health Law and Policy, Pune, India (see www.upsides.org for further information).

UPSIDES takes place over a 5-year period (2018–2022) and is divided into two phases, with different objectives and methods for each phase. The first 2 years (phase 1) focus on the development and piloting of a culturally appropriate peer support intervention [13]. The current paper focuses on the phase 2 lasts 3 years, when the finalised peer support intervention will be implemented and evaluated at multiple levels, including service user outcomes (psychosocial and clinical), PSW outcomes (empowerment and professional development), service outcomes (cost-effectiveness and return on investment), and implementation outcomes (adoption, sustainability and organisational change).

### Framework of the current study

**Fig. 1:**
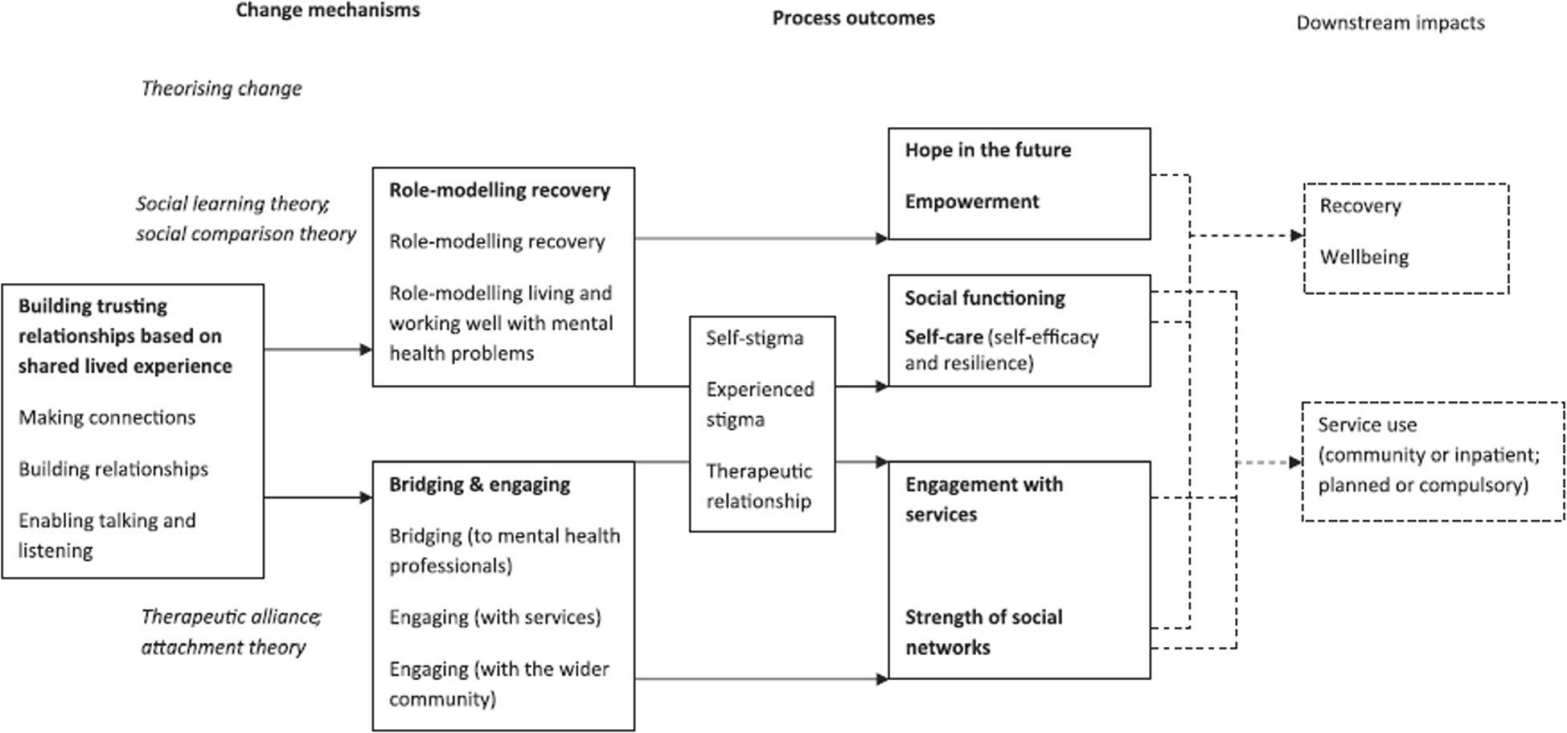
Change model underpinning peer worker interventions [14]

The theoretical framework underpinning the UPSIDES intervention is the model of change processes in mental health peer support developed by Gillard et al. [14] (Fig. 1). The model of Gillard et al., which is based on a large multisite qualitative study, provides a measurable set of outcomes expected to change in response to processes of peer support. These address active ingredients of peer support and relate to a range of process outcomes of peer working, including hope, social functioning and increased engagement with services, as well as downstream impacts in terms of recovery, well-being and service use.

**Fig. 2:**
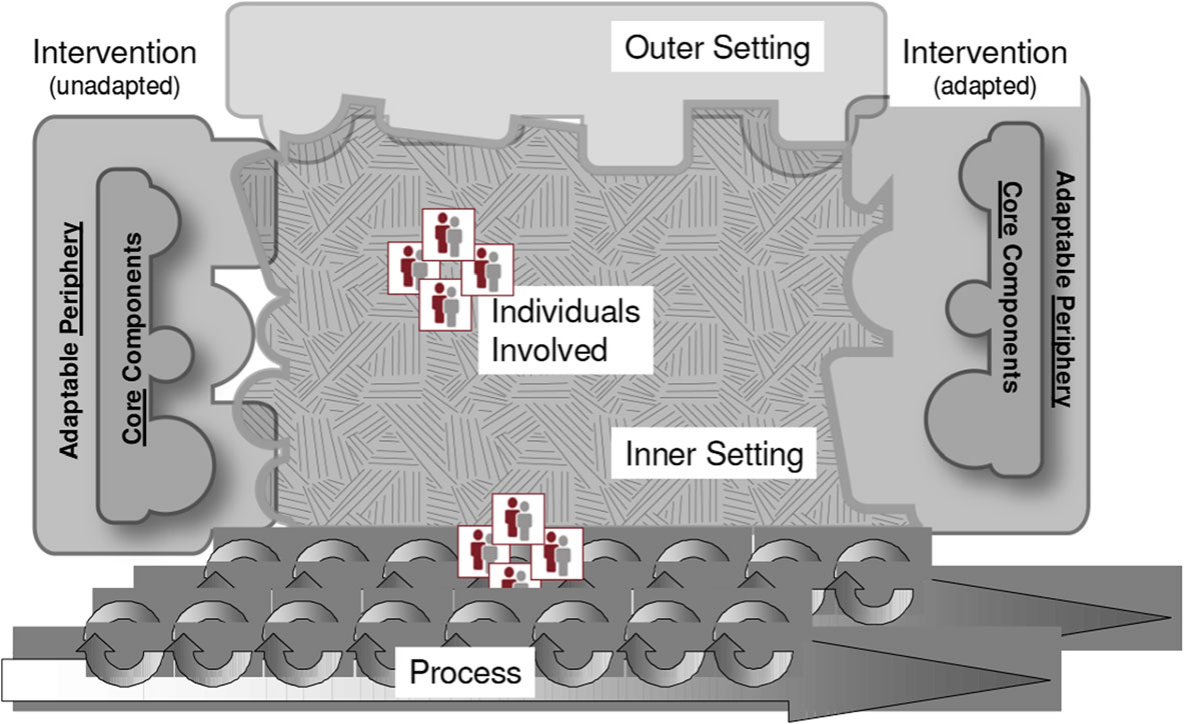
Major domains of the Consolidated Framework For Implementation Science [15]

UPSIDES research is also guided by the Consolidated Framework for Implementation Research (CFIR [15], Fig. 2) which promotes implementation theory development, helping to standardise investigation into and verification of what works, where, and why across multiple contexts. CFIR covers five major domains that can influence effective implementation: 1) intervention, 2) outer setting, 3) inner setting, 4) individuals involved and 5) process.

Building on results of formative research described elsewhere [13, 16, 17], UPSIDES peer support will be implemented across recruiting sites in line with an implementation manual, considering differences across study sites. The development of the manuals as well as their implementation and the overall study design will take into consideration the five CFIR domains by, for example, incorporating adaptations to local contexts, planning for organisational readiness, paying attention to the selection and involvement of relevant stakeholders, and allowing for a staged approach to continuously modify implementation as needed.

### Objectives and research question

The aim of UPSIDES-RCT is to explore the implementation and effectiveness of peer support delivered in a range of high-, middle- and low-income country settings. The main objectives are:

1. To evaluate the outcomes of delivering peer support, for service users, PSWs and organisations, through a multicentre, pragmatic, parallel-group, randomised controlled trial and additional qualitative methods.
2. To assess the value for money of peer support for persons with severe mental illness by carrying out a cost-effectiveness analysis.
3. To evaluate the process of implementing the UPSIDES peer support intervention, with special attention to differences in context across the study sites, using both quantitative and qualitative methods with PSWs, service users, mental health workers and wider stakeholders.

## Methods/design

This protocol covers the UPSIDES pragmatic parallel-group, multicentre, randomised controlled trial, cost-effectiveness analysis, and process evaluation, described further below. This study protocol adheres to the SPIRIT statement [18].

### Setting

Six of the eight UPSIDES collaborating institutions will host study sites:

1. Ulm, Germany (high income): catchment area of Ulm University’s Department of Psychiatry and Psychotherapy II at institutions which provide UPSIDES peer support.
2. Hamburg, Germany (high income): University Medical Centre Hamburg-Eppendorf and community services all over Hamburg, which provide UPSIDES peer support. Peer support has been implemented in Hamburg since 2007.
3. Kampala, Uganda (low income): the intervention will be provided at Butabika Hospital which is the main psychiatric hospital providing peer support in Uganda.
4. Dar es Salaam, Tanzania (low income): the intervention will be implemented at Muhimbili National Hospital at the Department of Psychiatry and Mental Health.
5. Be’er Sheva, Israel (high income): the intervention will be implemented at facilities in Israel which provide UPSIDES peer support.
6. Pune, India (lower middle income): the intervention will be implemented at the Hospital for Mental Health in Ahmedabad, Gujarat, which is a public mental health facility and has been implementing peer support since April 2015.

This is a sponsor-investigator multicentre trial. The coordinating centre is at Ulm. BP is the coordinating principal investigator (PI). PIs at UPSIDES-RCT study sites are BP (Ulm), CM (Hamburg), JN (Butabika), DS (Das es Salaam), GM (Be’er Sheva) and JK (Pune).

### Randomised controlled trial

UPSIDES-RCT is a pragmatic, parallel-group, randomised controlled multicentre trial which includes a waiting list, with four measurement points (t_0_ = baseline; t_1_ = 4 months; t_2_ = 8 months; t_3_ = 12 months).

#### Participants

Inclusion criteria: adult (age 18–60 years) at intake; mental disorder of any kind as main diagnosis established by case notes, staff communication or self-label; presence of severe mental illness (Threshold Assessment Grid, TAG [19] ≥5 points and illness duration ≥2 years); sufficient command of the host country’s language; capable of giving informed consent.

Exclusion criteria: main diagnosis of learning disability, dementia, substance disorder or organic brain disorder; cognitive impairment severe enough to make it impossible to give informed consent or complete study measures.

#### Processes, intervention and comparison

##### Intervention

All participants will receive treatment as usual. Participants allocated to the intervention group will additionally receive UPSIDES peer support, which is a formal service delivered by a trained person who has personal experience of mental illness to a person or a group of persons with a serious mental illness. UPSIDES PSWs will all be adults (age 18–60 years) who have experienced a mental illness and who have been stable or out of hospital for at least 3 months. PSWs will have progressed in their recovery beyond controlling symptoms to focusing on self-definition, growth and participation in the community. UPSIDES PSWs will be using these personal experiences, along with UPSIDES training and supervision, to facilitate, guide and mentor another person’s recovery journey [20]. Social support and recovery role modelling are central to UPSIDES peer support, while other elements may vary across sites depending on need and feasibility, for example, management, counselling, outreach, coaching and advocacy. The intervention has been developed by all UPSIDES partners through literature review and the adaptation of existing programmes [13]: ImROC training (UK [21]), Ex-IN curriculum (Germany [22]), Brain Gain projects (Uganda [23]), QualityRights (World Health Organization [24], India [25]), Healthy Options project (Tanzania [26]), Peer2peer (Europe [27]), and the Yozma Derech-Halev consumer-provider training (Israel [28]).

The peer support training has been manualised with a complementary workbook addressing underlying values and principles, specific skill training and preparing PSWs for tasks around recovery planning. The UPSIDES peer support training consists of 12 core modules: 1) understanding recovery and own recovery journey; 2) tree of life; 3) peer support; 4) communication; 5) recovery planning; 6) activating resources; 7) community and trialogue; 8) problem solving; 9) peer role description; 10) work preparation; 11) recovery groups; and 12) network. Training on local adaptations is provided via additional modules which consider context and address site-specific topics (social and environmental situation, resources in mental health care, stigma, rights and advocacy, trauma and disasters). An online training platform will facilitate exchange among trainers, PSWs and staff members at different study sites, helping to provide cross-site training and supervision while building an international PSW community.

UPSIDES peer support will be provided without hierarchy or judgement, taking into account principles of recovery-oriented care. UPSIDES PSWs will focus on: providing tangible supports; role modelling that recovery is possible; sharing personal experiences of mental health and ill-health; and promoting hope, and a sense of control and opportunities. Specific tasks include: initial assessment of strengths and resources to build on; practical support with daily life as needed (for example, accompaniment for appointments or activities); support during crises; and actively promoting recovery planning. Providing an additional recovery group setting is possible and recommended. UPSIDES peer support will be delivered for up to 6 months, with a minimum of three contacts. Weekly or biweekly meetings are recommended, but frequency may vary depending on the needs of service users, PSWs and study sites. The intervention manual provides additional materials to be used by the PSWs during the intervention. To support the implementation process, organisational readiness workshops are held across trial sites. Participants allocated to the control group (wait list) will start receiving the intervention after completion of follow-up (month 12). Criteria for discontinuing or modifying the intervention include change of content and dose of peer support or PSW in response to participant’s request, harms, or improving or worsening of illness.

##### Control

The control intervention is treatment as usual as provided at each of the respective UPSIDES-RCT study sites:

- Ulm: Psychiatric routine care in Germany is mainly provided by psychiatric hospitals, psychiatric outpatient clinics and office-based psychiatrists and psychotherapists. In addition, a broad spectrum of non-medical vocational, residential and psychosocial services are provided by vocational rehabilitation centres, community mental health care centres and different types of residential facilities. The Department of Psychiatry and Psychotherapy II at Ulm University is responsible for the provision of mental health care in a large catchment area in rural Bavaria (North and Middle Swabia, population 671,000). Multidisciplinary teams (psychiatrists, psychologists, social workers, nurses, occupational therapists) offer the full range of pharmacological and psychosocial interventions in a large inpatient unit, two day care units, an outpatient clinic, and a home treatment team (mobile crisis intervention). The Department collaborates closely with office-based psychiatrists and psychotherapists in the area.
- Hamburg: For routine psychiatric care in Germany, see Ulm above. The University Medical Centre Hamburg-Eppendorf is one of the largest hospitals in the City of Hamburg. The Department of Psychiatry and Psychotherapy has multidisciplinary teams who provide inpatient, outpatient and outreach (Crisis Resolution Teams, Assertive Community Treatment) mental health services in a large catchment area of several districts in Hamburg and cooperates closely with various service providers in the region.
- Butabika: Psychiatric services in Kampala are provided in the form of outpatient clinics at general hospitals as well as inpatient and outpatient care at the National Referral Hospital at Butabika. Physical health care, psychotherapies and social interventions and reintegration are provided at Butabika before service users are discharged back to their homes. Rehabilitation is provided at the Occupational Therapy Department at Butabika, as there are no public community-based mental health rehabilitation facilities. Treatment as usual will therefore comprise psychopharmacological as well as regular psychosocial care and occupational therapy provided on an inpatient or outpatient basis at Butabika Hospital.
- Dar es Salaam: Mental health services in Tanzania are decentralised, starting from primary care facilities which often serve as an entry point into the mental health system. At district hospitals, psychiatric nurses perform triaging, referring and refilling prescriptions for people with mental illness who are considered to be in a stable condition. People with severe and complicated mental illness are referred to tertiary care for specialised treatment. Tanzania experiences a considerable shortage of psychiatrists, and most psychiatrists work in tertiary care. In the Department of Psychiatry and Mental Health at Muhimbili National Hospital, Dar es Salaam, both inpatient and outpatient mental health services are available. Providers include psychiatrists, occupational therapists, social workers, psychiatric nurses and clinical psychologists. Mental health services provided include psychotherapy, psychosocial rehabilitation, vocational skills training, family intervention, cognitive enhancement therapy and psychoeducation.
- Be’er Sheva: Mental health care in Israel is provided by psychiatric hospitals, psychiatric outpatient clinics and office-based psychiatrists and psychotherapists. Psychiatric rehabilitation services are provided through the mandatory rehabilitation basket law and include a wide range of services in the community: vocational, residential and psychosocial services and programmes and community mental health care centres. The Yozma Derech-Halev programme specialises in supporting consumers who wish to work in rehabilitation or clinical services. It is independent of other services and supports consumers in numerous organisations who are employed in multidisciplinary mental health teams.
- Pune: Mental health care in India is broadly delivered through public and private mental health facilities with inpatient and outpatient departments. Community-based services are limited and generally based on a medical model of care. Services at public mental health facilities are provided at a nominal cost; however, these services are typically overburdened and under-resourced. Mental health care is often not available, not accessible, not acceptable and not of good quality, leading to a large treatment gap. The Hospital for Mental Health in Ahmedabad, Gujarat, caters to the city of Ahmedabad (8 million population approximately), with an inpatient facility of 300 clients, and outpatient unit serving 150 clients per day. Peer support volunteers are financially supported by the state at this site. Treatment as usual will therefore comprise psychopharmacological as well as regular psychosocial care and occupational therapy provided by the hospital on an inpatient or outpatient basis.

##### Outcomes

The primary outcome is social inclusion at t_2_ (8 months). This time point has been chosen because it is shortly after the intervention has been completed, but also allows time for changes in social inclusion to take place. Social inclusion is a key outcome in global mental health [29] and will be measured with the Social Inclusion Scale (SIS [30]) which is a service-user-reported measure with 16 items answered on a four-point Likert scale (“not at all”; “not particularly”; “yes a bit”; “yes definitely”). The total SIS score consists of the prorated sum over the 16 items, and can range from 16 to 64, with higher scores representing higher levels of social inclusion. The SIS has previously been shown to have adequate psychometric properties [30] which will be further investigated for the different language versions used in this trial.

Secondary outcomes are empowerment (using the Empowerment Scale, ES [31]), hope (using the HOPE scale [32]), recovery (using the Stages of Recovery, STORI-30 [33]), and health and social functioning (using the Health of the Nations Outcome Scales, HoNOS [34]). Established state-of-the-art translation guidelines [35] will be followed to translate and locally validate the standardised measures (including administration instructions) to be used, with special attention paid to the psychometric evaluation of the primary outcome. See Puschner et al. [13] for further details on translation.

##### Participant timeline

**Fig. 3:**
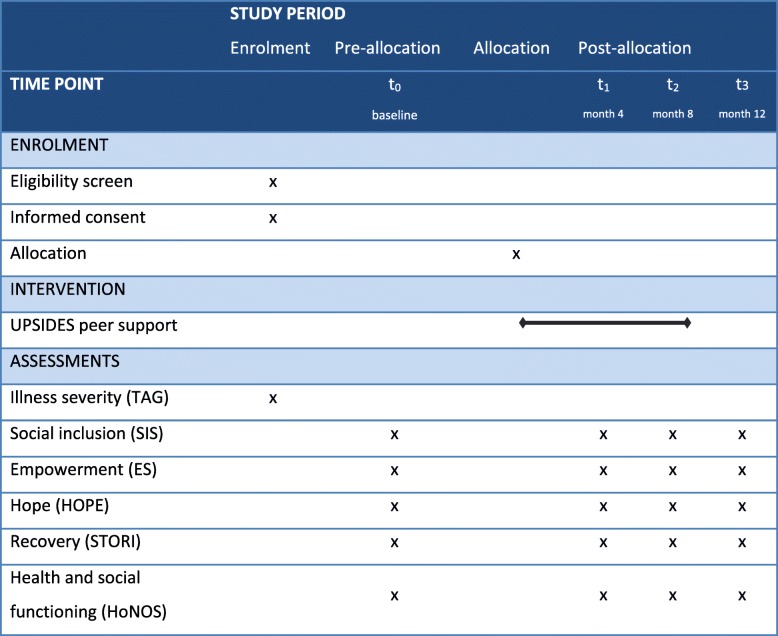
UPSIDES-RCT participant timeline. ES Empowerment Scale, HoNOS Health of the Nations Outcome Scales, HOPE Hope Scale, SIS Social Inclusion Scale, STORI Stages of Recovery, TAG Threshold Assessment Grid, UPSIDES Using Peer Support In Developing Empowering mental health Services

Figure 3 gives an overview of the trial’s participant timeline for the major stages of enrolment, allocation, and assessments in line with SPIRIT recommendations.

##### Recruitment and retention

Recruitment of study participants will vary among sites due to differences in mental health systems and services. Potential participants will be approached in various ways, including via outpatient/community mental health services, patient and carer organisations, local newspapers, social media, community leaders, and by word of mouth. The duration of the recruitment period is 12 months, starting in January 2020.

Once participants are enrolled, research workers will make every reasonable effort to follow them for the entire study period, including reminding them of the upcoming data collection and the benefits they will receive, and maintaining interest in the study through materials and mailings. Any deviations from the study protocol and the trial’s time plan (including withdrawal) or from the intervention and implementation manuals will be thoroughly documented.

##### Allocation

Participants will be randomly assigned to either the control or intervention group with a 1:1 allocation as per a computer-generated randomisation schedule stratified by site using permuted blocks of random sizes. The block sizes will not be disclosed to ensure concealment. Participants will be randomised by an independent unit (Institute for Medical Biometry and Epidemiology, Ulm, Germany). All participants who give consent for participation and fulfil the inclusion criteria will be randomised. Allocation concealment will be ensured as the service will not release the randomisation code until the participant has been recruited into the trial and the baseline assessments completed.

Randomisation will be requested by the staff member responsible for recruitment and clinical interviews from the Institute for Medical Biometry and Epidemiology, Ulm, by e-mail. The randomisation form includes site ID, participant ID, gender of participant, date of informed consent and approval of eligibility. The requesting research worker will get a response by mail within one working day. For participants in the intervention group, the research worker will inform the local PSW team and the participant. Throughout the study, the randomisation will be conducted by the Institute for Medical Biometry and Epidemiology, Ulm, to keep the data management and the statistician blind to the study allocation for as long as the data bank is open. The randomisation list remains with the Institute for Medical Biometry and Epidemiology for the duration of the study. Randomisation will therefore be conducted without any influence of the principal investigators or raters.

##### Blinding

Due to the nature of the intervention, neither participants nor PSWs can be blinded. Efforts will be made to blind staff collecting data during study visits (for example, by separating recruitment tasks from data collection) as far as is feasible. Researchers analysing study data will be blinded until the entire analysis has been completed, as described above.

##### Trial management and oversight

The Trial Management Group (TMG) consists of leads of study sites and is responsible for identification, recruitment and follow-up of study participants, data collection and adherence to the study protocol. The Trial Steering Committee (TSC) and Data Safety and Monitoring Board (DSMB) are oversight bodies that are independent of the sponsor and will be continuously informed of study progress, including data quality issues. The three members of the TSC will ensure that the trial is conducted in line with Good Clinical Practice. The TSC will have ultimate responsibility for the trial and will assume primacy over the DSMB and TMG. The TSC can prematurely terminate the trial (for example, in case of violations of patient safety). The DSMB will safeguard the interests of the study participants and monitor the data collected in the trial.

#### Statistical analysis and power calculation

Analyses will start once baseline data has been collected and cleaned. Descriptive statistics will be produced for all outcome measures, and outcome trajectories from t_0_ to t_3_ will be examined via exploratory analyses. The intervention’s effect on primary and secondary outcomes will be tested by means of random-effect regression models including a fixed group effect over time, allowing the inclusion of cases with incomplete (unbalanced) data across panels [36]. Post-hoc analyses will examine early (at t_1_) and late (at t_3_) effects for primary and secondary outcomes. All outcome analyses will be performed on an intention-to-treat basis. Per-protocol analyses will be part of the process evaluation (see below).

Sample size calculation was performed for testing whether the primary outcome (social inclusion) at t_2_ is affected by allocation. For six study sites, three time points, and estimated panel attrition of 10% at each time point, *N* = 558 participants (*N* = 93 per site) will be needed to detect a small effect size (0.25 SD units) with a power of 0.80 at a two-tailed significance level of 0.05. Sample size calculation was performed using the RMASS program (www.rmass.org) for a three-level mixed-effects linear regression model for the analysis of longitudinal data, assuming a linear effect over time, compound symmetry for error variance covariance, a person-level covariance (Int, Cov, Slope) of 0.300, 0.150, 0.100, and a centre-level covariance (Int, Cov, Slope) of 0.050, 0.025, 0.020.

### Cost-effectiveness analysis

#### Measures

Comprehensive societal costs of mental illness will be estimated for each participating country using an adapted version of the Client Sociodemographic and Service Receipt Inventory (CSSRI) [37] at t_0_ to t_3_. The CSSRI-UPSIDES will be adapted for use at all sites to assess mental health service use and productivity losses due to mental illness. If not already available, country-specific unit costs of health and social care services will be determined by investigating publicly accessible sources (price lists, catalogues of fees and charges), by expert interviews or by calculations of staff and capital costs for services used, drawing on the principles recently developed by the Global Health Cost Consortium in their reference case for global health costing [38]. All costs will be converted into USD ($) using purchasing power parities [39]. Quality-adjusted life years (QALYs) will be estimated using the EQ-5D 3 L at t_0_ to t_3_ as used in most multinational cost-utility studies [40] and being available in the languages of all study sites. As country-specific population-based value sets are not available for most study sites, QALYs will be estimated on the basis of the European EQ-5D visual analogue scale for all countries [41].

#### Data analysis

Incremental cost-utility ratios will be estimated for each country by calculating the ratio between the average difference in total costs of illness and the average QALY difference over 12 months. Stochastic uncertainty of incremental cost-effectiveness ratios will be assessed by non-parametric bootstrapping with 2,000 replications. The maximum willingness-to-pay (MWTP) necessary to cover 95% of the incremental cost-effectiveness ratio variance will be estimated by means of the cost-effectiveness acceptability curves [42]. In order to take into account the economic differences between participating countries, country-specific MWTP thresholds suggested by Woods et al. [43] and Leech et al. [44] will be applied.

Net monetary benefit regression models with random effects for the UPSIDES treatment, fixed country effects and fixed treatment by country interaction effects will be computed to estimate the impact of country-specific differences on cost utility [45]. Individual net monetary benefit values will be computed for each study participant by multiplying the individual country-specific QALYs with MWTP thresholds ranging from $0 to $50,000 (steps will be defined on the basis of results from the incremental cost-utility analysis and subtracting country-specific individual costs [45]). Marginal effects of the net monetary benefit regression models will be computed to estimate country-specific net monetary benefits for defined MWTP thresholds. Robust standard errors will be estimated to account for skew distribution of net benefit values [46, 47]. Results from the primary data incremental cost-utility analysis will be used to develop a decision tree model [48] to simulate cost utility and health budget impact of the UPSIDES intervention for each country over a 5-year time frame [49]. The sensitivity analysis will consider variance of programme coverage and equity of service access [50].

### Process evaluation

Participants in the process evaluation include service users, PSWs, mental health workers and other key informants involved in the UPSIDES intervention. PSWs and service users will be recruited from the participants in the randomised controlled trial (see above). Purposive sampling strategies will be applied for mental health workers and key informants. Mental health workers will be recruited from multidisciplinary teams who cooperate with PSWs. Key informants will be local stakeholders with relevant expertise relating to implementation of peer support work, including clinicians and managers who employ PSWs.

#### Quantitative component of process evaluation

Information on key process variables will be collected from different stakeholders (Table 1). Data analysis will apply random-effect regression models and structural equation modelling including process variables as moderators or mediators of effect. Additional multivariate analyses will be carried out to analyse the effect of fidelity including dose (number, frequency and duration of peer support sessions), recorded by routinely administered monitoring and evaluation (M&E) forms, and implementation outcomes.

**Table 1:** UPSIDES-RCT process measures

Variable	Scale	Number of items	Raters	Sample size total/per site	Timing^a^		
Measures as part of randomised controlled trial data assessments					t_0_	t_1_	t_2_
Experiences of peer support	Brief INSPIRE [51]	5	SU	279/47		x	x
Fidelity	UPSIDES peer support fidelity scale	32 25	SU PSW	279/47 60/10		x	
Other measures^a^					T_0_	T_1_	T_2_
Motivations, competencies and relationship characteristics	Recovery-oriented peer provider work-role model and prototype measure (ROPP) [52]	29	PSW	60/10	x	x	x
Empowerment	Empowerment Scale (ES) [31]	28	PSW	60/10	x	x	x
Recovery	Stages of Recovery (STORI-30) [33]	30	PSW	60/10	x	x	x
Recovery orientation	Recovery Self-Assessment (RSA) [53]	36	Mental health workers	30/5	x		x
		36	Key informants	12/2	x		x

*PSW* peer support worker, *SU* service user, *t*_*0*_ baseline, *t*_*1*_ 4 months, *t*_*2*_ 8 months

^a^Measurement points for other measures based on the entire duration of the intervention: T_0_ = baseline or earlier (at the start of the intervention); T_1_ = month 12 (intermediate); T_2_ = month 24 or earlier (at the end of the intervention)

#### Qualitative component of process evaluation

The purpose of the qualitative component is to gain a deep multilayered contextual understanding of the impact and experiences of the UPSIDES peer support intervention among stakeholders. This component of the process evaluation includes four different qualitative studies, each focusing on a different target group: 1) service users’ experiences of the peer support intervention; 2) occupational development of PSWs; 3) practitioners’ experiences of peer support; and 4) key informants’ experiences of peer support.

A common overall methodology shared by each of these studies is described below and followed by detailed information on each of the individual studies. Table 2 gives an overview of qualitative studies which are part of the process evaluation.

**Table 2:** Overview of qualitative studies

No.	Participants	Method	Sample size per site	Time point	
				T_0_	T_2_
1	Service users	Interviews	6–8		x
2	Peer support workers	Focus groups	2 groups (3–7)		x
3	Mental health workers	Focus groups	1 group (6–8)	x	x
4	Key informants	Focus groups	4–6	x	x

Measurement points for other measures based on the entire duration of the intervention: T_0_ = baseline or earlier (at the start of the intervention); T_2_ = month 24 or earlier (at the end of the intervention)

##### Overall methodology

Each study will follow the following steps: 1) provide verbal and written introduction to the study (participant information) and obtain written consent (consent form); 2) conduct and electronically record interviews or focus groups according to flexible topic guides; 3) collection of sociodemographic data; 4) transcribe audio recording verbatim and pseudonymise; 5) translate field notes and transcripts into English, if necessary; and 6) analyse data thematically through grouping and structuring of relevant themes to address key issues as per study objectives.

Data analysis will involve reviewing all field notes, reading and re-reading all the transcripts for familiarisation, consensual coding and generating themes. The data analysis will be conducted by a core group, supported by partners at each study site involved in data collection as needed. Data analysis will adhere to the following steps: 1) based on 1–2 interviews with participants, the task lead will develop preliminary codes and categories (themes); 2) preliminary codes and categories (themes) will be reviewed by the core group and commented on; 3) modifications of the coding tree will be commented on by the core group (the final coding tree will be discussed and revised as necessary until consensus is reached); 4) subsequent inclusion of interviews and modifications of sub-categories (themes); 5) reviewing, refining and defining themes; and 6) validation of the coding structure with each local site.

A code book will be developed using the data from all sites, which will then be shared across sites for review, discussion and standardisation. The pre-determined code book will be used to develop notes (data managed into units of information that cover broad categories with grouping of relevant emerging themes of importance). Each site will comment on the emerged themes, and the core analysis group will finalise the themes.

The study-specific core group will decide on the software to be used for data management and text retrieval (for example, QSR International’s NVivo 12 qualitative software, MAXQDA). The focus will be on the semantic content of the data (as responses to semi-structured questions). Quality will be improved by the use of multiple analysts to ensure that a range of perspectives informs the interpretation of the data; the use of verbatim quotes for each theme, to ensure the interpretation is as close to the data as possible; and local validation to maximise cross-cultural validity of the coding framework.

#### Service users’ experiences of peer support intervention

Objectives: In order to identify factors that contribute to the impact of peer support on service users, this study will explore service users’ views on positive/negative effects of peer support, and factors which moderate the positive/negative effects of peer support on social inclusion, (self) stigmatisation, empowerment, hope, recovery, and illness concepts. By using an open approach, the study will shed light on service users’ everyday experiences of the intervention beyond what is already known or expected regarding the mechanisms of impact, including active ingredients and conditions for optimal experiences as well as barriers and contextual influences.

Participants: Following a mixed-method approach, 6–8 service users from the intervention group at each site will be purposively selected at the end of intervention using their quantitative outcome data to include those with and without benefit (1:1) of peer support in terms of social inclusion and experiences of peer support. Participants will be selected based on their ratings on the SIS [30] and Brief INSPIRE [51] and grouped into ‘low responders’ (with a combination of low scores on the SIS and low scores on Brief INSPIRE) and ‘high responders’ (with a combination of high scores on the SIS and high scores on Brief INSPIRE).

Methods: Since an open approach allows for a deeper insight into personal systems of meaning among the interviewees [54], semi-structured interviews will be conducted at the end of the intervention to capture service users’ experiences with and subjective effects of peer support, and their attitudes towards peer support. Interviewers will use a flexible interview guide including 6–8 open questions to capture everyday experiences with peer support (for example, talks and activities), subjective appraisal of the positive/negative effects of peer support (for example, self-esteem, self-stigma and knowledge) and attitudes towards provision of peer support within mental health settings (for example, contextual factors, barriers and facilitators).

#### Peer support workers’ occupational development

Objective: To evaluate the impact of training and implementation on the occupational development of PSWs, to identify the views of PSWs on what contributes to or hinders successful implementation of peer support, and the attitudes of PSWs towards the benefits and challenges of the intervention. The study will address the gender-specific views of the PSWs on their occupational roles, facilitators and barriers, and resources and needs.

Participants: Two focus groups (one male, one female) per site with 3–7 participants in all six study sites, for 12 focus groups in total.

Methods: To allow for comparison, as well as to capture the diversity of PSW characteristics and activities across countries, focus group guidelines will include two parts. First, questions for PSWs across all sites focusing on: 1) experiences of PSWs and perspectives in their trained roles, including training and working as PSWs in this project; 2) the views of PSWs on personal benefit and challenges when using lived experiences in their role as a PSW; 3) the views of PSWs regarding effects on service users; and 4) barriers, facilitators and needs for successful implementation of peer support. Second, context-sensitive questions depending on a specific country’s stage of implementation and cultural characteristics focusing on obstacles and challenges of the PSW intervention at study sites. At study sites where the peer support model is new (for example, Ulm and Dar es Salaam), questions will focus on inner and outer setting factors relevant to the implementation of peer support (for example, local values and norms, power dynamics, organisational facilitators and barriers to implement peer support and to develop the occupational roles of PSWs). At study sites with more advanced implementation (for example, Be’er Sheva and Butabika), questions will focus on the experiences of PSWs in assimilation and sustainability of their role, benefits and challenges with the use of lived experiences (for example, self-disclosure and boundaries), and organisational facilitators and barriers to enhancing and sustaining peer support and to developing the occupational roles of PSWs (for example, financial arrangements).

#### Practitioners’ experiences of peer support

Objective: To investigate 1) the impact on the multidisciplinary team of PSWs joining and working in the team (“impact on team culture”); 2) the impact on the individual clinician of working alongside a PSW as a colleague (“impact on clinician”); 3) implementation issues that helped or hindered the successful implementation of PSWs in the team (“implementation”); and 4) the impact perceived by the clinician on service users who received peer support (“impact on service user”).

Participants: Mental health workers working in the same team as an UPSIDES PSW.

Methods: 6–8 mental health workers in each site will take part in a focus group before and after the intervention. A replacement clinician will be identified for follow-up if a baseline focus group attendee is no longer available (for example, because they have moved jobs). Purposive sampling will be used to maximise diversity in terms of age, gender and years since qualifying. Data will be collected using an open focus group guide based on an implementation scale developed through a previous systematic review [16]. This covers elements of organisational culture, training, role clarity, resourcing, access to a peer network and other factors essential for successful peer support implementation. In addition, at baseline and follow-up, focus group participants will be asked to complete a 16-item questionnaire (Mental Illness: Clinicians’ Attitudes Scale [55]) to assess changes of stigmatising attitudes towards people with mental illness after working with PSWs.

#### Key informants’ experiences of peer support

Objectives: To explore the practical consequences, promises and challenges of peer support from the perspectives of key informants and to assess implementation outcomes in line with the CFIR [15]. This study will use focus groups to explore stakeholders’ views on: 1) perceived changes/difficulties prior to the intervention versus implementation outcome; 2) barriers and facilitators for successful implementation of peer support in the given institution; and 3) the need for changes or alterations to make the intervention work effectively in a specific context.

Participants: Key informants will be defined as experts with specific authorities and/or responsibilities in regard to the implementation processes at study sites. Experts are supposed to have specific contextual (organisational) knowledge about implementation of peer support in mental health settings (for example, policy makers and representatives of service user, provider and funder organisations). Experts will be selected as appropriate to the local structures of mental health services.

Methods: At each site, focus groups with 4–6 key informants will be held at the beginning and end of the intervention. Purposive sampling strategies will be applied based on the local knowledge of study site researchers about relevant key informants. Topics covered will include acceptability, appropriateness and feasibility, to be guided by the CFIR interview guide tool [56], including characteristics at the levels of the intervention (for example, advantages, risks/costs and cultural adaptability), the outer setting (for example, barriers and facilitators for meeting service users’ needs, policies and incentives), the individual (for example, knowledge and values), and the intervention processes (for example, obstacles and facilitators during planning, execution and evaluation).

### Ethics and dissemination

Obtaining informed consent in a manner appropriate to the local context is fundamental to the ethical conduct of research and is of particular importance. Each potential participant in this research project will be clearly informed prior to consent of the goals and the possible risks of the project, and will be given the possibility to either refuse to enter or withdraw consent without any adverse consequences. We will ensure that study information is understood by all study participants, and that the focus on improving health care practice is conveyed. A simple written description of the intervention will be provided, along with an informed consent form that describes what is required for participation (for example, time commitment and what is being asked of participants). It will be explained to participants what happens to their data and recordings once the research project is completed (i.e. that data will continue to be stored safely at the study sites). Information about the pros and cons of study participation will be also given by trained UPSIDES research workers as part of this process. The UPSIDES research workers will be trained to assess decisional capacity based on the four basic elements related to decisional capacity described by Appelbaum [57]. This assessment ensures that the participant understands the information relevant to their participation and demonstrates that they can retain this information, weigh it up, and communicate their decision. The name of a person to contact if a participant wants to withdraw from the study will appear in the informed consent form.

Prospective participants who are literate will be given a copy of the information sheet, and will have the opportunity to ask questions before signing the consent form in writing. Potential participants who are illiterate will be read the information sheet by the UPSIDES research worker and given the opportunity to ask questions, and may sign the consent form with a thumb print in the presence of a witness. Each participant will be given a copy of the written informed consent on a piece of paper to keep for themselves.

Participants will receive reimbursement for study participation and for their expenses for travelling to the research centre as per site-based policies. There is no anticipated harm and provision of post-trial care. This trial does not involve collecting biological specimens for storage. The protocol, site-specific informed consent forms (local language and English versions) and other requested documents will be reviewed by the UPSIDES Ethics Advisor and the local ethical review bodies (Institutional Review Boards (IRBs)/Ethics Committees (ECs)).

#### Confidentiality and dissemination

Only research staff and representatives of the local EC will have access to study data. Data on paper will be stored in lockable locations. Electronic data will be stored in password-protected locations. Data transferred electronically will be pseudonymised and encrypted. At baseline entry, each participant will receive a study ID. This study strictly complies will European Union guidelines on data protection including General Data Protection Regulation (GDPR; Regulation 2016/679).

Research results will be disseminated in open-access, peer-reviewed journals and shared through oral and poster presentations at international conferences. All resources (policy briefs, research summaries, training tools, manuals, and so forth) will be uploaded to an online knowledge management platform. Anonymised data will be made publicly available for further analyses after final predetermined publications in a public repository in line with Pilot on Open Research Data in Horizon 2020.

#### Protocol amendments

Any modifications to the protocol which may impact the conduct of the study, potential benefit of the participants or may affect participant safety—including changes of study objectives, study design, patient population, sample sizes, study procedures, or significant administrative aspects—will require a formal amendment to the protocol. Such an amendment will be agreed upon by TMG and TSC, and presented to UPSIDES Ethics Advisor and the local ethical review bodies (IRBs/ECs).

## Discussion

Given the scarcity of mental health workers in low- and middle-income countries and a recent paradigm shift towards mental health recovery in high-income countries, involvement of peers in mental health care has the potential to improve and to transform mental health services in low-, middle- and high-resource settings.

This study addresses several knowledge gaps and limitations in previous randomised controlled trial designs and is in line with calls to continue to research and improve the quality of studies on peer support in mental health [6, 58]. First, evaluating the development of an effective peer intervention using a longitudinal design combining both quantitative and qualitative methods has been previously recommended [59]. Second, the qualitative component of the study will yield insights into the complex processual aspects of implementing peer support; for example, exploring the role of a PSW and what do peer providers do to successfully create change, both for recipients and for organisations? Third, the randomised controlled trial addresses ethical concerns regarding the use of comparison groups in intervention studies for vulnerable populations by adopting a wait-list design. Finally, as much as possible, this study protocol follows best practices for the minimisation of research biases, while considering differences in local contexts [60].

Previous reviews of randomised trials of peer support have highlighted the heterogeneity of interventions and outcome measures used in many studies, positing that lack of standardisation may contribute to null or mixed results in this area of research (for example, see [6]). The current study addresses these concerns by developing and testing a peer support intervention based on core elements common to all participating study sites, and further investigates processes of change that have previously been identified as relevant to mental health peer support interventions. Furthermore, it will focus on social inclusion as the primary outcome, alongside other outcomes directly related to personal recovery, in line with established definitions of mental health peer support [8, 59, 61]. In addition, the study will include mental health and economic outcomes reported to be missing from many studies of peer support according to previous reviews [6, 58].

### Limitations

A multicentre, randomised controlled trial can prove challenging, particularly in low-resource settings and given differences in mental health care structures. For example, aligning timelines for tasks can be difficult when working around infrastructural and contextual challenges. Ethical review protocols and committee recommendations vary across settings, requiring flexibility within the overall framework without compromising on quality and fidelity to the model. While overall there has been much involvement in the development of the intervention by users and PSWs, the study planning was more heavily reliant on researchers in academic institutions. It is also necessary to plan for the high likelihood of attrition among peer staff who may need to take sick-leave or drop out unexpectedly. Proper support and accommodation for PSWs will be put in place to maximise retention and sustainability.

## Conclusions

UPSIDES-RCT is the first multisite, randomised controlled trial to study peer support in a range of low-, middle- and high-income countries, addressing a number of geographical, methodological and other knowledge gaps in international research on mental health peer support. Conducting an international implementation research study will allow the identification of universal versus local/contextual elements of the intervention, contributing to the evidence base for peer support and its theoretical underpinnings in other similar settings. By explicitly studying process as well as outcomes, UPSIDES asks not just whether peer support works in these settings, but how, in order to provide practical guidance on the implementation and scale-up of peer support in different settings. Ultimately, UPSIDES aims to inform mental health policy, implementation and practice to ensure that the perspectives and potential contributions of people with lived experiences are taken into account.

## Data Availability

Any data required to support the protocol can be supplied on request.

## Abbreviations

CFIR: Consolidated Framework for Implementation Research
DSMB: Data Safety and Monitoring Board
PSW: Peer support worker
QALY: Quality-adjusted life year
SIS: Social Inclusion Scale
TMG: Trial Management Group
TSC: Trial Steering Committee
UPSIDES-RCT: Using Peer Support In Developing Empowering mental health Services Randomised Controlled Trial
